# Use of Quadruple Therapy in the Management of Hypertension: A Systematic Review of Randomized Controlled Trials

**DOI:** 10.3390/medicina61040764

**Published:** 2025-04-21

**Authors:** Khalid A. Alnemer

**Affiliations:** Department of Internal Medicine, Faculty of Medicine, Imam Mohammad Ibn Saud Islamic University (IMSIU), Riyadh 13317, Saudi Arabia; kaalnemer@imamu.edu.sa

**Keywords:** quadruple, hypertension, combination, systolic blood pressure, diastolic blood pressure

## Abstract

*Background and objectives*: Hypertension remains a leading global cause of cardiovascular morbidity and mortality, with suboptimal blood pressure (BP) control despite available treatments. Monotherapy often fails to achieve target BP, necessitating combination therapies. Quadruple low-dose combination therapy (quadpill) has emerged as a potential strategy to enhance efficacy while minimizing side effects. This systematic review evaluates the effectiveness and safety of quadpill therapy compared to standard monotherapy and dual therapy. *Methods*: A systematic search was conducted in PubMed, Web of Science, and Scopus from inception till January 2025 for randomized controlled trials (RCTs) investigating quadruple therapy in hypertensive patients. Studies comparing quadpill therapy with monotherapy, dual therapy, or placebo were included. Data on BP reduction, achievement of target BP, and adverse events were extracted and analyzed. The Cochrane Risk of Bias tool (RoB-2) was used to assess study quality. *Results*: Five RCTs were included in the current systematic review. Quadpill therapy resulted in greater reductions in systolic BP (SBP and diastolic BP (DBP) compared to monotherapy and dual therapy across all time points. The proportion of patients achieving target BP (<140/90 mmHg) was significantly higher in the quadpill group. The safety profile was favorable, with adverse event rates comparable to those in monotherapy and dual therapy groups. Notable adverse effects included mild dizziness, edema, and biochemical alterations (elevated fasting glucose and uric acid levels), but these did not lead to significant treatment discontinuation. *Conclusions*: Quadruple low-dose combination therapy is a promising approach for improving BP control while maintaining a favorable safety profile. Early initiation of quadpill therapy could mitigate treatment inertia and improve long-term cardiovascular outcomes.

## 1. Introduction

Hypertension is the predominant cardio-cerebrovascular disorder globally and frequently coexists with additional cardiovascular risk factors, resulting in harm to vital organs [1,2]. Hypertension is the foremost risk factor for mortality worldwide, responsible for 10.8 million deaths in 2019 [3], and it imposes a considerable economic burden. Nevertheless, despite its widespread occurrence and effects, the knowledge, treatment, and control rates of hypertension are unsatisfactory, with certain statistics indicating these metrics to be as low as 50.0%, 38.1%, and 11.1%, respectively [4].

The American College of Cardiology/American Heart Association (AHA/ACC) and European Society of Cardiology (ESC) guidelines set different blood pressure (BP) targets but share a focus on individualized treatment. The AHA/ACC define hypertension as BP ≥130/80 mmHg, recommending a target of <130/80 mmHg for most adults, including high-risk patients, while emphasizing lifestyle changes and early intervention. The ESC suggests a more refined approach, with a target systolic BP of 120–129 mmHg for most patients if tolerated, introducing an “elevated BP” category (120–139/70–89 mmHg) to better identify cardiovascular risk. Tight BP control typically refers to achieving lower BP targets, such as systolic BP between 120 and 129 mmHg. While this approach can further reduce the risk of cardiovascular events, it may increase the likelihood of adverse effects like hypotension or syncope. Therefore, it is essential to balance the benefits and risks, considering patient-specific factors such as age, comorbidities, and treatment tolerance [5,6].

Hypertension management is suboptimal, with control rates ranging from 6% to 30% observed across various groups [7]. Partially, this may involve monotherapy, wherein less than 40% of a Caucasian population may achieve a goal BP (<140/90 mm Hg) using typical angiotensin-converting enzyme inhibitors, beta-blockers, calcium channel blockers, or diuretics [8]. Numerous reasons contribute to inadequate BP management, including low adherence rates, intricate recommendations advocating multiple up-titration steps, and treatment inertia. The majority of treated patients receive just monotherapy [9], which exhibits minimal efficacy even at elevated doses [10]. Moreover, the mounting evidence indicates the advantages of more aggressive BP reduction underscores the necessity for novel therapeutic options that are both more effective and tolerable [11,12].

Mild hypertension is often managed with monotherapy; however, guidelines increasingly indicate that most individuals with severe or resistant hypertension will necessitate two or more antihypertensive medications to meet the necessary targets [13,14]. If blood pressure remains elevated despite dual therapy, stepping up to triple therapy becomes essential. Yet, some patients continue to experience uncontrolled hypertension despite the use of three different antihypertensive agents. In such complicated situations where blood pressure cannot be adequately controlled with single or dual therapy, the prescription of a quadpill—a combination of four low-dose antihypertensive agents—becomes a necessary and effective strategy [15,16]. For patients with moderate to severe hypertension, particularly those with multiple cardiovascular risk factors, target organ damage, or resistant hypertension, a more aggressive approach is necessary as well. Polypharmacy may correlate with a heightened incidence of adverse medication reactions and potentially diminished patient adherence [17,18]. Evidence indicates that adherence to a single-pill combination antihypertensive is superior to simultaneous two-pill therapy [19].

Low-dose, single-pill combinations exhibit significant potential to address these obstacles [15,16]. Dose–response studies of particular drugs demonstrate that optimal benefits are attained, and the majority of negative effects are mitigated at low dosages [15,20]. The quadpill notion refers to a single pill that amalgamates four distinct antihypertensive drugs, each administered at one-quarter of the normal dosage for hypertension. Prior brief studies indicated that the quadpill exhibited superior BP-lowering efficacy compared to regular dose monotherapy [21], and significant advantages above placebo [16]. A larger trial using a triple half dose compared to standard care in Sri Lanka yielded encouraging outcomes [22].

## 2. Materials and Methods

By adherence to the Preferred Reporting Items for Systematic Reviews and Meta-Analyses (PRISMA) guidelines [23] (Supplementary Materials), this systematic review was performed by searching for all eligible publications on PubMed, Web of Science, and Scopus from inception till January 2025. The search strategy was based on two primary keywords and their corresponding Medical Subject Headings (MESH) terms: “Hypertension” AND “Quadruple” OR “Quadpill”.

### 2.1. Eligibility Criteria and Screening

Following the search, articles were imported into Rayyan to facilitate the filtering process [24]. The preliminary screening employed titles and abstracts, succeeded by an exhaustive full-text evaluation to determine eligibility. We included hypertensive patients treated with quadruple therapy compared with monotherapy, dual therapy, or placebo regarding the effect on BP control. The research design specifications included randomized controlled trials (RCTs). Case reports, observational studies, and reviews were excluded.

### 2.2. Data Extraction

The baseline characteristics of the studies, including research design, gender, age, sample size, interventions, follow-up, and dose, were extracted using Microsoft Excel sheets. Outcome data, encompassing the effect on BP, SPB, and DBP, and adverse outcomes, were also extracted.

### 2.3. Risk of Bias Assessment

Two distinct authors conducted a risk of bias assessment, and any inconsistencies were sent to a third author for resolution. We utilized diverse assessment tools in accordance with the study design. For RCTs, we utilized the Cochrane risk of bias tool (Rob-2), which includes five domains, each with a set of questions. The results are then included into a visual to determine one of three levels of bias: low risk, moderate concern, or high risk. A study is considered to have a low overall risk of bias if all five domains are assessed as exhibiting a low risk of bias. If any domain raises concerns, the study is deemed to have potential bias issues. If any domain demonstrates a significant risk of bias or multiple domains exhibit issues, the study is categorized as having a high risk of bias [25].

### 2.4. Data Synthesis

We systematically reviewed the included articles and a narrative synthesis of data was conducted to present the efficacy and safety parameters in addition to a summary of findings of the included studies as presented in results and tables.

## 3. Results

### 3.1. Searching and Screening

**Figure 1:**
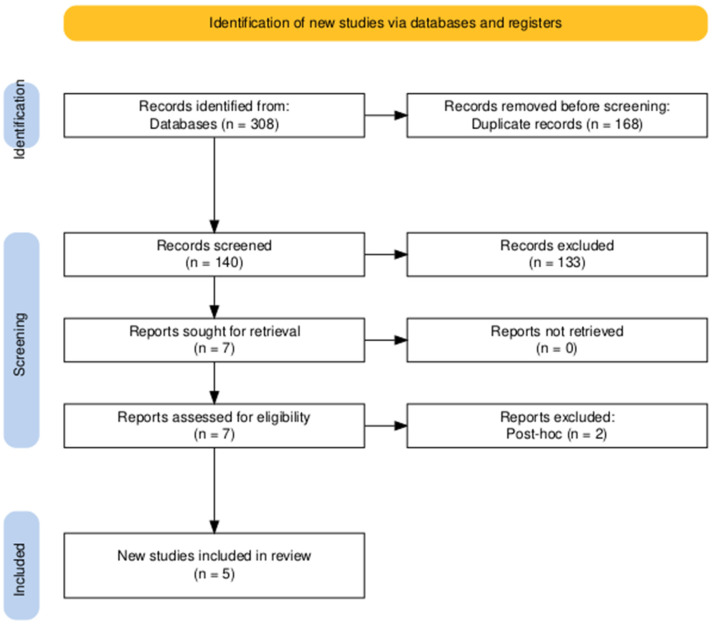
PRISMA flow diagram of searching and screening.

The conducted search strategy yielded 308 entries, of which 168 were classified as duplicates. After evaluating the titles and abstracts of the remaining 140 papers, seven studies satisfied the criteria for evaluation of the complete text. In conclusion, five publications were deemed suitable for inclusion in the final systematic review [16,21,26,27,28] (Figure 1).

### 3.2. Risk of Bias Assessment

**Figure 2:**
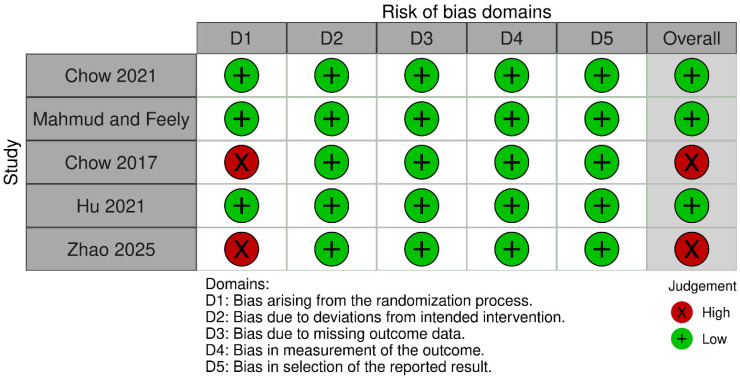
Risk of bias assessment of RCTs using Rob-2 [16,21,26,27,28].

According to Rob-2, two RCTs had high risk of bias, while three RCTs were deemed to have low risk of bias (Figure 2).

### 3.3. Baseline Characteristics of Study Participants

The baseline characteristics of study participants were assessed across multiple treatment arms, including various fixed-dose quadruple therapy regimens, standard monotherapy, dual therapy, and placebo groups. These characteristics include sample size, mean age, male distribution, and follow-up duration, providing a comprehensive overview of the study population. We included five RCTs encompassing two crossover trials. The sample sizes varied across studies, with the largest cohort consisting of 300 participants receiving quadruple therapy involving irbesartan, amlodipine, indapamide, and bisoprolol. The smallest cohort involved 21 participants receiving irbesartan, amlodipine, hydrochlorothiazide, and atenolol combination therapy. The mean age across all groups ranged from approximately 43.88 years (SD 10.31) to 59 years (SD 11), indicating a diverse population of hypertensive patients. The proportion of male participants varied among studies, with the highest male representation observed in the study by Hu et al. Follow-up durations varied across studies, with some lasting as long as one year, particularly for large cohort studies examining long-term BP effects. Shorter follow-up durations of one to three months were noted for other included trials (Table 1).

### 3.4. Systolic Blood Pressure (SBP) Reduction

In all intervention groups, significant reductions in SBP were observed compared to monotherapy or placebo, reinforcing the benefits of combination therapy in managing hypertension. The fixed-dose quadruple combination therapy achieved greater SBP reductions across all follow-up points (6, 12, 26, and 52 weeks) compared to standard treatments, with reductions sustained over time. Among patients receiving a combination of irbesartan 37.5 mg, amlodipine 1.25 mg, indapamide 0.625 mg, and bisoprolol 2.5 mg, SBP was significantly lower than in those receiving irbesartan 150 mg alone. The observed reductions in SBP were clinically significant, demonstrating that lower doses of multiple antihypertensive agents in combination are more effective than higher doses of a single agent. Moreover, 24 h, daytime ambulatory, nighttime ambulatory, and office SBP were lower in the half-dose quadruple treatment (irbesartan 75 mg, metoprolol 23.75 mg, amlodipine 2.5 mg, and indapamide 1.25 mg) compared with the standard-dose dual treatment (irbesartan 150 mg and amlodipine 5 mg). Moreover, a combination of reserpine 0.1 mg, dihydralazine 12.5 mg, hydrochlorothiazide 12.5 mg, and triamterene 12.5 mg showed a high reduction in SPB compared with placebo.

### 3.5. Diastolic Blood Pressure (DBP) Reduction

Similar trends were observed for DBP, with intervention groups showing consistently lower DBP values compared with comparators. Combination therapies yielded more substantial reductions in DBP than individual drug regimens, with effects sustained throughout the study duration.

### 3.6. Achievement of Blood Pressure Targets

The percentage of patients achieving BP targets (<140/90 mmHg) and tight BP control (<120/80 mmHg) was higher in low-dose quadruple therapy compared with monotherapy confirming its superior efficacy in achieving optimal BP control. At each time point, the intervention group consistently outperformed standard monotherapy, reinforcing the early and sustained impact of combination therapy. The data suggest that an early initiation strategy of combination therapy could prevent long-term cardiovascular complications associated with poorly controlled hypertension.

### 3.7. Safety and Adverse Events

Across all treatment arms, adverse event rates were comparable between intervention and comparator groups, indicating the tolerability of low-dose combination therapy. Common adverse events included dizziness, pedal edema, muscle cramps, hypersensitivity reactions, and gastrointestinal complaints, but these were mild and did not result in significant treatment discontinuation. No significant differences in serious adverse events were noted between groups, supporting the safety profile of fixed-dose combination therapies as the quadruple therapy did not increase the rates of drug discontinuation compared with the standard therapy. Apart from significant increases in fasting blood glucose and blood uric acid in the half-dose quadruple group compared with dual therapy, and although metabolic changes (elevated fasting glucose, increased uric acid levels) linked with diuretics, electrolyte imbalances (hypokalemia, hyponatremia), and effects on renal function are the main concerns in the use of quadruple therapy, no other adverse events or changes in laboratory values differed significantly between the two treatments, which shows the safety of quadruple therapy use. Moreover, the reserpine-based combination was associated with decreased potassium levels compared with the placebo, which warrants further monitoring (Table 2).

## 4. Discussion

This study confirmed that a fixed-dose quadruple combination was more effective in lowering BP than standard monotherapy approaches, with sustained effects over time. Early initiation of combination therapy resulted in superior BP control with an acceptable safety profile, supporting its role as a first-line strategy for hypertension management. These findings align with previous research suggesting that quarter-dose therapy across multiple antihypertensive classes provides additive benefits while maintaining tolerability.

The use of quadpill therapy, a fixed-dose combination of four antihypertensive agents at low doses came from the principle of using combination therapies for hypertension at low doses that avoid side effects and maintain benefits. This was shown to be effective in resistant cases that failed dual or triple therapies in addition to being safe [15,16]. The use of quadpill aligns with the principles of major hypertension guidelines, including those from the ESC, ACC/AHA, and International Society of Hypertension (ISH), which emphasize early and effective blood pressure control. While these guidelines advocate for combination therapy, particularly dual or triple therapy for resistant cases, they do not yet explicitly endorse quadpill therapy as a primary treatment strategy [29,30,31,32]. The potential benefits of the quadpill include improved adherence, rapid blood pressure reduction, and minimized side effects due to lower individual drug doses. However, significant barriers exist to its widespread adoption, including limited regulatory approval, cost considerations, and physician preference for stepwise titration, which allows for individualized dose adjustments. Additionally, concerns about flexibility in modifying treatment regimens may make some clinicians hesitant to transition from traditional approaches. Despite these challenges, the quadpill’s ability to enhance treatment efficacy suggests that further clinical evidence and future guideline updates could support its broader integration into hypertension management.

The evidence of advantages from reducing BP beyond conventional objectives [31,33,34] have exacerbated the challenges associated with implementation. The alteration in objectives in the 2017 US guideline update elevated the percentage of US people with managed hypertension exhibiting BP values exceeding the goal from 39% to 53% [35]. In light of prior corroborative evidence [35], the SPRINT study [12] has significantly impacted global recommendations. Despite the SPRINT population commencing with a greater number of prescriptions and a potentially elevated risk, the BP reductions attained in both the QUARTET [28] and SPRINT intervention groups were comparable, as were the mean baseline BP values. In QUARTET [28], the drop in BP was predominantly accomplished in a single step—this simplicity represents a significant potential advantage. An implementation study is essential to ascertain the optimal integration of ultra-low dosage combinations into existing global treatment algorithms [36].

Inadequate BP regulation is a worldwide issue [9,37]. Commencing treatment with a dual-drug combination therapy has been recommended [38] as a more efficacious approach to swiftly attain BP regulation while minimizing clinic visits [39]. Chow et al. utilized the same foundational concepts but advanced the concept by commencing therapy with numerous ultra-low-dose medicines within a single capsule [40]. In contrast to current methods of BP-lowering therapy, the use of a single quadruple combination capsule is expected to yield greater reductions in BP than increasing the dosage of monotherapy, as doubling the dose of antihypertensive medications from half or standard dosage results in only an additional 1–2 mm Hg decrease in SBP or DBP [15]. Furthermore, a quadpill strategy may mitigate treatment inertia associated with both clinicians and patients by diminishing dependence on stepwise titration, which is infrequently accomplished in practice. A quadpill also accounts for individual variability in response to various agents by offering a combination with multiple mechanisms of action. Enhanced adherence is likely due to both reduced pill burden [41] and the administration of lower doses to mitigate side effects [15].

Achieving target BP early is recognized to reduce cardiovascular risk and improve prognosis [42,43,44,45]. Intensive management of patients with grade 1 and 2 hypertension could prevent 803,000 cardiovascular incidents annually and enhance 1.2 million quality-adjusted life years relative to current practices [46], resulting in significant socioeconomic advantages. The PURE trial observed that fewer than one-third of hypertensive patients reached target BP following the initiation of monotherapy [9]. In comparison to monotherapy, initiating combination therapy for antihypertensive treatment enhanced the degree of BP reduction and decreased the time required to achieve target BP [47,48,49], especially in patients with grade 1 hypertension [50,51,52].

In recent years, certain researchers have posited the hypothesis that low-dose multidrug combinations (≥3) yield superior antihypertensive effects and reduced side effects during initial treatment, conducting preliminary investigations in this domain [16,28,53,54,55]. This challenges the conventional notion that current antihypertensive regimens commence with a combination of two drugs, which is further supported by the 2023 ESH hypertension guidelines that reference the quadpill concept [29].

The research by Zhao et al. [55] reinforced the conclusion that a small dose of quadruple medications was more successful in reducing BP than standard-dose dual medications. Zhao et al. [55] indicated that half-dose quadruple therapy decreased SBP by 4.72 mmHg more than standard-dose dual therapy, a reduction that is less than the QUARTET study [28], in which quadruple quarter-dose therapy reduced SBP by 6.9 mmHg more than standard-dose monotherapy. This aligns with the prevailing belief that the combination of two distinct drugs is more efficacious than increasing the dosage of a single treatment. In recent years, an increased time in target range (TTR) has been correlated with a reduced risk of mortality from all causes and significant adverse cardiovascular events [56,57,58]. Zhao et al. [55] conducted a retrospective analysis of this parameter and discovered that a small dose of triple medications might greatly enhance TTR.

Attaining control of BP continues to be a significant difficulty [59,60]. Poor BP control can be attributed to various factors, including societal and health system variables, inadequate adherence, and clinical inertia. An approach that achieves a sustained reduction of 7 mm Hg in SBP is anticipated to result in an 11% decreased risk of ischemic heart disease and an 18% decreased risk of stroke and heart failure over the long term. Significant opportunity exists in low-income and middle-income countries, where most individuals with hypertension globally live, with fewer than one-third receiving treatment, and approximately 30% of those treated achieving BP control [61,62]. The initial application of ultra-low-dose combination therapy may substantially enhance BP regulation in these contexts, provided that issues related to availability, price, and health system integration are addressed [60,63]. This strategy’s description should be included in hypertension guidelines, while its use is currently constrained by the availability of appropriate goods.

Zhao et al. [55] indicated that the half-dose quadruple combination resulted in a higher incidence of adverse events concerning fasting blood glucose and blood uric acid, potentially attributable to beta-blockers and diuretics [64,65,66,67]. The occurrence of gout resulting from the quadruple combination remained nonexistent, despite elevated uric acid levels. Moreover, a half-dose quadruple combination may decrease blood potassium and salt levels while increasing creatinine and urea to some degree, maybe linked to diuretics [68,69]. Nonetheless, these minor alterations lacked clinical significance as compared to the baseline [55].

### 4.1. Strengths and Limitations

The study synthesizes data from multiple RCTs, including crossover trials, to provide a comprehensive evaluation of quadruple therapy. The study addresses a significant global health issue—poor hypertension control—and provides practical insights into improving treatment adherence through single-pill combinations. While larger trials (e.g., QUARTET) provide strong evidence, some included RCTs had small sample sizes, limiting generalizability. This highlights the need for larger, high-quality RCTs to strengthen the evidence base. Additionally, a publication bias assessment (e.g., Egger’s test or funnel plot) should be considered in future meta-analyses (that could not be conducted due to the variability in and limitation of data) to determine whether smaller studies were omitted, potentially skewing the results. Differences in drug combinations, dosages, and follow-up durations introduce variability, affecting direct comparability. Heterogeneity assessment statistics (e.g., I^2^ values) should be provided for key outcomes in future studies. Potential sources of heterogeneity, such as variations in study populations, treatment regimens, and BP measurement techniques, should also be discussed and their confounding effects should be adequately addressed. While short-to-medium-term safety profiles are favorable, longer follow-up including cardiovascular events, mortality reduction, and adherence rates is needed to assess potential cumulative adverse effects and adherence strategies.

### 4.2. Implications for Future Practice

Quadruple therapy should be considered for early adoption in hypertension guidelines, particularly for patients with poor BP control on monotherapy or dual therapy. Moreover, the use of quadruple therapy improves patient’s adherence to treatment compared with conventional titration methods, which are shown to be incompatible. Therefore, real-world settings should be a focus of future studies to validate this. Future studies should explore patient-specific factors (e.g., age and comorbidities) to optimize drug selection within the quadruple combination framework. Given minor metabolic changes, routine biochemical monitoring should be incorporated into clinical practice when using this regimen.

## 5. Conclusions

This systematic review reinforces the superior efficacy of low-dose quadruple combination therapy in achieving rapid and sustained BP control compared with standard monotherapy or dual therapy. The findings align with emerging recommendations advocating for combination therapy as a first-line approach in hypertension management. Notably, the strategy enhances adherence while maintaining a favorable safety profile, positioning it as a promising alternative to traditional stepwise titration methods. With appropriate guideline integration and clinical adoption, this approach has the potential to revolutionize hypertension treatment, improving global BP control and reducing cardiovascular morbidity and mortality. Combining drugs from different classes at low doses can result in synergistic effects, leading to more effective and consistent blood pressure reduction compared with monotherapy or even dual therapy. This is particularly important in the elderly, who are at higher risk of cardiovascular complications due to poorly controlled hypertension. Additionally, the quadpill simplifies the treatment regimen by reducing the number of pills taken daily, which can improve medication adherence. This is especially beneficial in older patients who may already be managing complex medication schedules due to comorbidities. Furthermore, using lower doses of each drug tends to minimize the risk of dose-related side effects, making the therapy more tolerable.

## Figures and Tables

**Table 1:** Baseline characteristics of the included studies.

Study ID	Design	Intervention	Comparator	Doses		Sample Size		Age, Mean (SD)		Male, n (%)		Follow-Up
				Intervention	Comparator	Intervention	Comparator	Intervention	Comparator	Intervention	Comparator	
Chow 2021 [28]	RCT	Irbesartan, Amlodipine, Indapamide, and Bisoprolol	Irbesartan	Irbesartan 37.5 mg, Amlodipine 1.25 mg, Indapamide 0.625 mg, and Bisoprolol 2.5 mg	Irbesartan 150 mg	300	291	58 (12)	59 (11)	178 (59)	178 (61)	1 year
Mahmud and Feely 2007 [21]	RCT	Amlodipine, Atenolol, Bendroflumethiazide, and Captopril	Amlodipine, Atenolol, Bendroflumethiazide, or Captopril	Amlodipine 1.25 mg, Atenolol 12.5 mg, Bendroflumethiazide 0.625 mg, and Captopril 12.5 mg	Amlodipine 5 mg, atenolol 50 mg, Bendroflumethiazide 2.5 mg, or Captopril 50 mg	22	86	50 (2)	49.8 (2.8)	15 (68.2)	55 (64)	1 month
Chow 2017 [16]	Crossover randomized trial	Irbesartan, Amlodipine, Hydrochlorothiazide, and Atenolol	Placebo	Irbesartan 37.5 mg, Amlodipine 1.25 mg, Hydrochlorothiazide 6.25 mg, and Atenolol 12.5 mg	NA	21		58 (11)		10 (48)		1.5 and 1 month
Hu 2021 [26]	RCT	Reserpine, Dihydralazine, Hydrochlorothiazide, and Triamterene	Placebo	Reserpine 0.1 mg, Dihydralazine 12.5 mg, Hydrochlorothiazide 12.5 mg, and Triamterene 12.5 mg	NA	30	30	47.1 (13)	46 (13.1)	25 (83.3)	17 (56.7)	3 months
Zhao 2025 [27]	Crossover randomized trial	Irbesartan, Metoprolol, Amlodipine, and Indapamide	Irbesartan and Amlodipine	Irbesartan 75 mg, Metoprolol 23.75 mg, Amlodipine 2.5 mg and Indapamide 1.25 mg	Irbesartan 150 mg and Amlodipine 5 mg	90		43.88 (10.31)		67 (74.4)		1 month

SD: standard deviation, RCT: randomized controlled trial.

**Table 2:** Outcomes measured across the included studies.

Study ID	Effect on SBP	Effect on DPB	Effect on BP	Adverse Events	Summary of Findings
Chow 2021 [28]	Unattended automated and office SBP was lower in the intervention compared with the control group at 6, 12, 26, and 52 weeks.	Unattended automated and office DBP was lower in the intervention compared with the control group at 6, 12, 26, and 52 weeks.	Incidence of achievement of BP target (office BP < 140/90 mm Hg) and tight BP target (office BP < 120/80 mm Hg) was higher in the intervention group compared with the control group at 6, 12, 26, and 52 weeks.	No significant difference in adverse events was observed between the intervention and control including dizziness, pedal edema, muscle cramps, hypersensitivity, gastrointestinal complaints, musculoskeletal complaints, and headache.	A strategy with early treatment of a fixed-dose quadruple quarter-dose combination achieved and maintained greater BP lowering compared with the common strategy of starting monotherapy. This trial demonstrated the efficacy, tolerability, and simplicity of a quadpill-based strategy.
Mahmud and Feely 2007 [21]	The percentage reduction in SBP was greater with the combination than the individual drugs.	The percentage reduction in DBP was greater with the combination than the individual drugs.	The reduction in MAP with the combination was significantly greater than that with individual agents. More patients achieved a BP of 140/90 mmHg with the combination than any individual drug.	NR	A low-dose combination of 4 agents representing 4 classes of standard antihypertensive agents was more efficacious than a standard single dose of each agent individually
Chow 2017 [16]	24 h, daytime ambulatory, nighttime ambulatory, and office SBP were lower in the intervention compared with placebo	24 h, daytime ambulatory, nighttime ambulatory, and office DBP were lower in the intervention compared with placebo	The incidence of office BP less than 140/90 mmHg was significantly higher in the intervention group compared with placebo.	No significant difference was obtained between intervention and placebo in adverse events	The findings of this trial suggest that the benefits of quarter-dose therapy could be additive across classes and might confer a clinically important reduction in BP.
Hu 2021 [26]	Quadruple treatment showed high reduction in SPB compared with placebo	Quadruple treatment showed high reduction in DPB compared with placebo	The control rate of hypertension was higher in the combination than placebo group.	No significant difference in adverse events and no serious adverse events occurred but serum potassium concentration was significantly lower in the quadruple combination group than the placebo group.	The study showed that the single-pill quadruple combination of reserpine, dihydralazine, hydrochlorothiazide, and triamterene effectively reduced BP in patients with grade 1 hypertension, with some known side effects of the component drugs, such as the changes in plasma norepinephrine and serum potassium. The finding on the changes in plasma serotonin warrants further investigation in studies involving this drug and other classes of antihypertensive drugs.
Zhao 2025 [27]	24 h, daytime ambulatory, nighttime ambulatory, and office SBP were lower in the half-dose quadruple treatment compared with standard-dose dual treatment	24 h, daytime ambulatory, nighttime ambulatory, and office DBP were lower in the half-dose quadruple treatment compared with standard-dose dual treatment	NR	Apart from significant increases in fasting blood glucose and blood uric acid in the half-dose quadruple group, no other adverse events or changes in laboratory values differed significantly between the two treatments.	Initiating treatment with half-dose quadruple combination therapy was more effective in lowering BP than standard-dose dual therapy. The safety of both combinations was comparable.

BP: blood pressure, SBP: systolic blood pressure, DBP: diastolic blood pressure, NR: not reported.

## Supplementary Materials

The following supporting information can be downloaded at: https://www.mdpi.com/article/10.3390/medicina61040764/s1, Reference [70] is cited in the supplementary materials.
